# Structural Evolution of TIR-Domain Signalosomes

**DOI:** 10.3389/fimmu.2021.784484

**Published:** 2021-11-17

**Authors:** Surekha Nimma, Weixi Gu, Natsumi Maruta, Yan Li, Mengqi Pan, Forhad Karim Saikot, Bryan Y. J. Lim, Helen Ying McGuinness, Zannati Ferdous Zaoti, Sulin Li, Sneha Desa, Mohammad Kawsar Manik, Jeffrey D. Nanson, Bostjan Kobe

**Affiliations:** School of Chemistry and Molecular Biosciences, Institute for Molecular Bioscience and Australian Infectious Diseases Research Centre, University of Queensland, Brisbane, QLD, Australia

**Keywords:** protein structure, protein-protein interactions, axon degeneration, cell-death signaling, signaling by cooperative assembly formation (SCAF), innate immunity, plant disease resistance, toll/interleukin-1 receptor/resistance protein

## Abstract

TIR (Toll/interleukin-1 receptor/resistance protein) domains are cytoplasmic domains widely found in animals and plants, where they are essential components of the innate immune system. A key feature of TIR-domain function in signaling is weak and transient self-association and association with other TIR domains. An additional new role of TIR domains as catalytic enzymes has been established with the recent discovery of NAD^+^-nucleosidase activity by several TIR domains, mostly involved in cell-death pathways. Although self-association of TIR domains is necessary in both cases, the functional specificity of TIR domains is related in part to the nature of the TIR : TIR interactions in the respective signalosomes. Here, we review the well-studied TIR domain-containing proteins involved in eukaryotic immunity, focusing on the structures, interactions and their corresponding functional roles. Structurally, the signalosomes fall into two separate groups, the scaffold and enzyme TIR-domain assemblies, both of which feature open-ended complexes with two strands of TIR domains, but differ in the orientation of the two strands. We compare and contrast how TIR domains assemble and signal through distinct scaffolding and enzymatic roles, ultimately leading to distinct cellular innate-immunity and cell-death outcomes.

## Introduction

**Figure 1:**
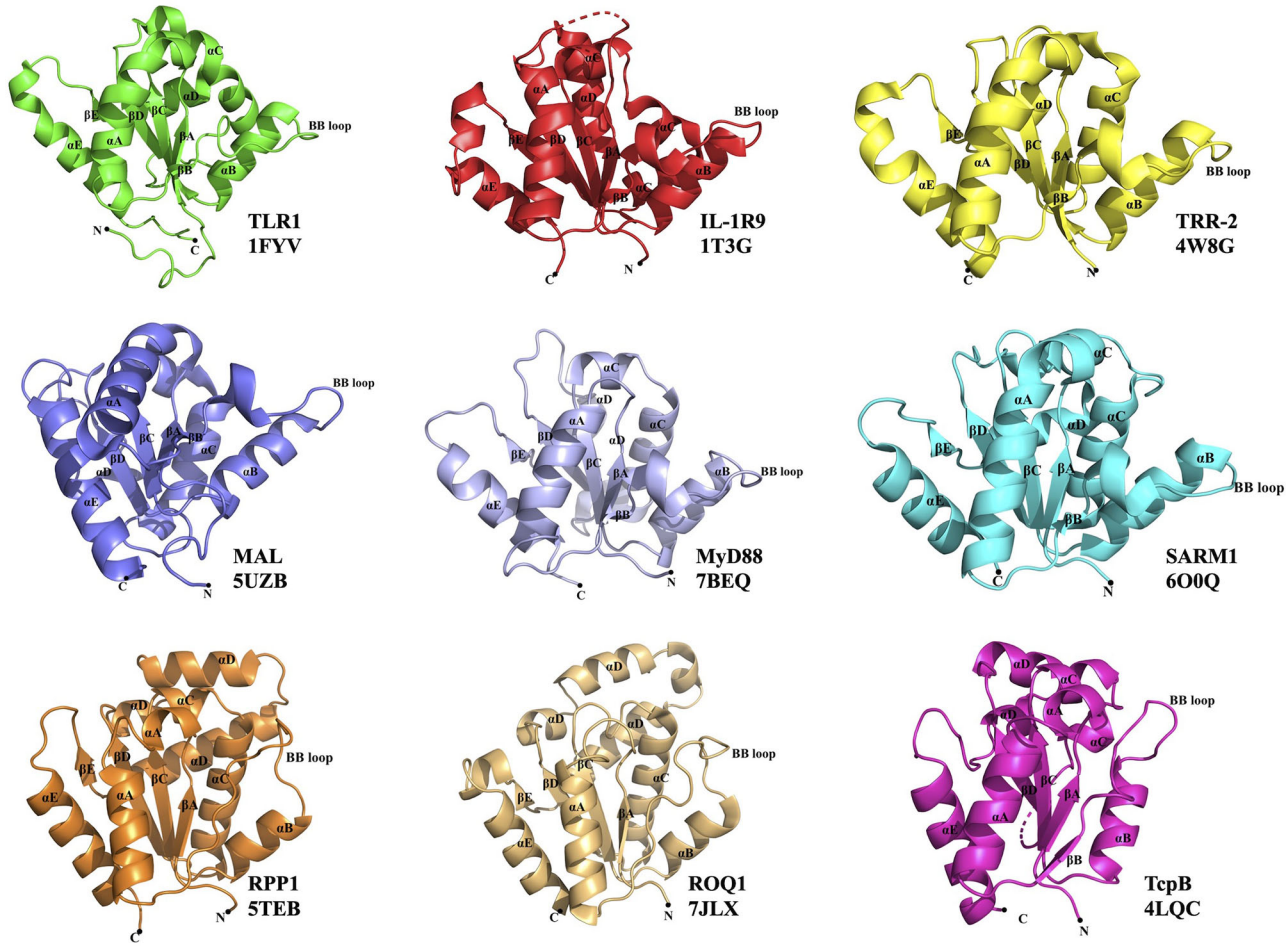
Representative TIR domain structures. TIR domains of the human (TLR1, IL-1R9, MAL, MyD88 and SARM1), lower metazoan *Hydra magnipapillata* (TRR-2), plant (RPP1 and ROQ1) and bacterial (TcpB) proteins with their corresponding PDB IDs are shown. All the TIR domains show a central core of five β-strands (βA–βE) surrounded by five α-helices (αA–αE). The functionally important BB-loop in each TIR is labeled.

TIR (Toll/interleukin-1 receptor/resistance protein) domains are cytoplasmic domains found in both eukaryotic and prokaryotic proteins that are involved in innate-immunity and cell-death pathways. They consist of 135–160 residues and typically display a five-stranded parallel β-sheet (strands βA–βE) surrounded by five α-helices (αA–αE) (**Figure 1**) (1, 2). TIR-domain functions are governed by weak and transient interactions. They predominantly function through homotypic interactions, including self-association or association with other TIR domains, to create scaffolds that facilitate signal transduction, leading to immune and cell-death responses (3, 4). The mechanism of signaling employed has been described as SCAF (signaling by cooperative assembly formation) (5–7). SCAF involves the assembly of higher-order complexes - signalosomes or “supramolecular organizing centers” (SMOCs) (8). In the case of SCAF, receptor activation through activating ligand binding induces receptor oligomerization, which in turn nucleates recruitment and oligomerization of adaptor proteins, and subsequently the recruitment and oligomerization of effector enzymes that can be activated through proximity-induced mechanisms in the resulting signalosome.

**Figure 2:**
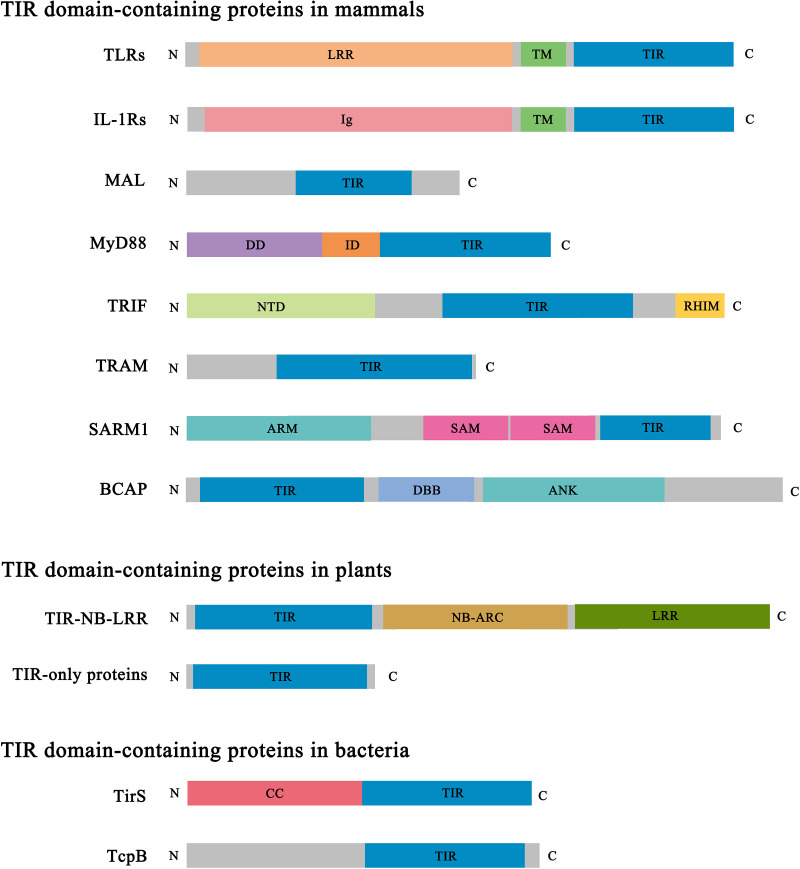
Domain architecture of representative TIR domain-containing proteins. TIR, Toll/interleukin-1 receptor/resistance protein; LRR, leucine-rich repeat; TM, transmembrane; Ig, immunoglobulin; DD, death domain; ID, intermediate domain; NTD, N-terminal domain; RHIM, RIP (receptor-interacting protein) homotypic interaction motif; ARM, armadillo-repeat motif; SAM, sterile alpha motif; DBB, Dof (Drosophila downstream of fibroblast growth factor receptor)/BCAP/BANK (B cell scaffold protein with ankyrin repeats); ANK, ankyrin repeat; NB-ARC, nucleotide-binding adaptor shared by APAF-1 (apoptotic protease-activating factor 1), R proteins and CED-4 (cell death protein 4); CC, coiled coil.

In mammals, TIR domains are found in Toll-like receptors (TLRs), interleukin-1 receptors (IL-1Rs) and cytoplasmic adaptor proteins, such as MyD88 (myeloid differentiation primary response gene 88) and MAL (MyD88 adaptor-like protein) (**Figures 1**, **2**) (2). TLRs and IL-1Rs are pattern recognition receptors (PRRs) that recognize evolutionarily conserved pathogen-associated molecular patterns (PAMPs) and endogenous danger-associated molecular patterns (DAMPs) released by dying or damaged cells (3, 4). Upon activation, TLR TIR domains dimerize, creating an intracellular TIR-domain signaling scaffold, which then recruits TIR domain-containing adaptor proteins that activate further downstream signaling (e.g. recruiting IL-1R-associated kinases (IRAKs) and activating the transcription factor NF-κB [nuclear factor kappa-light-chain-enhancer of activated B cells]), to induce inflammatory responses through the production of proinflammatory cytokines and programmed cell-death responses (3, 4).

In contrast to the scaffolding protein-protein interaction function described above, the TIR domain-containing protein SARM1 (sterile alpha and TIR motif containing 1) has been found to cleave NAD^+^ (nicotinamide adenine dinucleotide) and NADP^+^ (nicotinamide adenine dinucleotide phosphate) and initiate axon degeneration, in a process also dependent on the self-assembly of SARM1 TIR domains (**Figure 2**) (9–11). The dual role of TIR domains, serving as scaffolds or as enzymes, relies in part on the different assembly mechanisms of the proteins, leading to functional specificity.

Similar to mammals, plants also have a complex immune system to protect themselves from pathogen invasion. To restrict pathogen infection, the intracellular innate immune receptors called NLRs (nucleotide-binding domain (NBD)/leucine-rich repeat (LRR) receptors) directly or indirectly detect pathogen effector proteins *via* their C-terminal LRR domains and activate defense responses, including localized programmed cell death, in a process termed hypersensitive response (HR) (**Figure 2**) (12). The central NBD (usually called NB-ARC – see **Figure 2**) is important for the oligomerization of these proteins into a signalosome called the “resistosome” upon activation. TIR domains are found at the N-termini of a large group of NLRs (TIR domain-containing NLRs, TNLs), as well as TIR-only proteins, and truncated NLRs lacking LRR domains (**Figure 2**) (12, 13). It is unclear if some plant TIR domains serve scaffolding functions like the canonical mammalian TIR domains; however, plant TIR domains self-associate and display the enzymatic function of cleaving NAD^+^, sharing similarities with the function of SARM1 (10, 14). In agreement, plant TIR domains exhibit similar assembly mechanisms as SARM1 (10, 15, 16).

TIR domains are also found in bacteria and archaea. Structures and functions of bacterial and archaeal TIR domains and the corresponding proteins are poorly characterized, compared to their animal and plant counterparts, and are not the focus of the current review. Bacterial TIR domains are found in a wide range of domain architectures and domain types, which indicates diverse functional roles (**Figure 2**) (17). Some bacterial TIR domains, including TcpC (*Escherichia coli*), TirS (*Staphylococcus aureus*), PumA (*Pseudomonas aeruginosa*) and TcpB (*Brucella melitensis*), are linked to bacterial pathogenicity (18, 19). TirS and TcpC possess NAD^+^-nucleosidase activity (20), but so do bacterial TIR domains from non-pathogenic bacteria, suggesting roles in bacterial physiology (20, 21). Recent studies have also shown that the NAD^+^-nucleosidase activity of the bacterial TIR domains is linked to bacterial antiviral defenses (22–24).

Here, we review structural information available for various TIR domains, focusing on the nature of the different assemblies they form and their corresponding functional roles. Based on the available structural and functional evidence, we observe a correlation of TIR-domain assembly with their specific functional roles. In this respect, they fall into two different groups, the “scaffold” assemblies involved in innate-immunity signaling, and “enzyme” assemblies leading to NAD^+^ cleavage associated with cell-death signaling.

## Scaffold TIR-Domain Assemblies

This group comprises the TIR domains that undergo self-association to form a scaffold, which facilitates nucleation-controlled cooperative recruitment of other TIR domain-containing proteins and signal transduction (employing a SCAF mechanism) (5–7, 25). The group includes TIR domains from mammalian membrane receptors (TLRs and IL-1Rs), as well as those from the cytoplasmic adaptor proteins. A number of crystal structures of TIR domains from this group have been determined, but the structural basis of their self-assembly only became clear through the structural studies of higher-order structures reconstituted for the adaptors MAL and MyD88 (26, 27).

### TLRs and IL-1Rs

Ten TLRs are present in humans and found either on the cell surface (TLR1, TLR2, TLR4, TLR5, TLR6 and TLR10) or in intracellular endosomal compartments (TLR3, TLR4, TLR7, TLR8 and TLR9) (28). TLRs are characterized by an extra-cytoplasmic LRR domain, a transmembrane domain, and an intracellular TIR domain (**Figure 2**). When activated by their ligands, they mostly function as homodimers, but TLR2 functions as a heterodimer with either TLR1 or TLR6. A considerable amount of structural information is available on their LRR domains (29, 30), and even on the full-length TLR3 and TLR7 in complex with the membrane chaperone UNC93B1 involved in TLR trafficking (although the TIR domains could not be visualized in this case) (31). Crystal structures of the TIR domains of TLR1 (1), TLR2 (1, 32), TLR6 (33) and TLR10 (34) have been reported. These TIR domains, when expressed as separate proteins, are all monomeric in solution under the conditions tested. The characteristic BB-loop (connecting the βB strand and the αB helix) has been shown to play an important role in signaling, as a naturally occurring mutation (P712H) in this loop in TLR4 makes it non-responsive to the PAMP lipopolysaccharide (LPS) (35). Other mutations in this loop were also shown to abolish signaling in numerous TIR domains, including TLR4 (36) and TLR7 (37). Downstream signaling involves the recruitment of the cytoplasmic adaptors MyD88 (for all TLRs except TLR3), MAL (as a bridging adaptor for MyD88 in the case of TLR4 and TLR1/2/6), TRIF (TIR domain-containing adaptor protein-inducing interferon β; for TLR3) and TRAM (TRIF-related adaptor molecule; as a bridging adaptor for TRIF in the case of TLR4).

There are also ten IL-1Rs (IL-1R1 to IL-1R10) in humans, characterized by extracellular immunoglobulin domains, a transmembrane domain, and an intracellular TIR domain (except for IL-1R2) (38) (**Figure 2**). IL-1Rs recognize and bind specific IL-1 family cytokines, which leads to recruitment of an accessory receptor chain. The downstream signaling mechanism is similar to that of TLRs, involving recruitment of MyD88, IRAKs and activating NF-κB to induce inflammatory responses (39). The IL-1R TIR domains are structurally less well characterized, with the majority of the available structural data limited to the complexes involving the ectodomains and their corresponding ligands (40). The only available structure of a TIR domain corresponds to that from IL-1R9 (IL-1RAPL1, IL-1R accessory protein like 1) (41). However, IL-1R9 is not a classical signaling IL-1R; it does not activate NF-κB, but has been reported to be involved in trans-synaptic signaling (42).

### Cytoplasmic TLR Adaptor Proteins

TLRs and IL-1Rs require adaptor proteins for signaling. Six adaptor proteins have been identified in humans: MyD88, MAL, TRIF, TRAM, SARM1 and BCAP (B-cell adaptor for phosphoinositide 3-kinase) (28) (**Figure 2**). MyD88, MAL, TRIF and TRAM are the principal signaling adaptors in this pathway (43), while SARM1 and BCAP have been reported to negatively regulate TLR signaling (44–46). Crystal and/or NMR structures are available for the TIR domains from all these proteins (2, 5, 10).

### Structures of Higher-Order Assemblies of MAL and MyD88 TIR Domains

**Figure 3:**
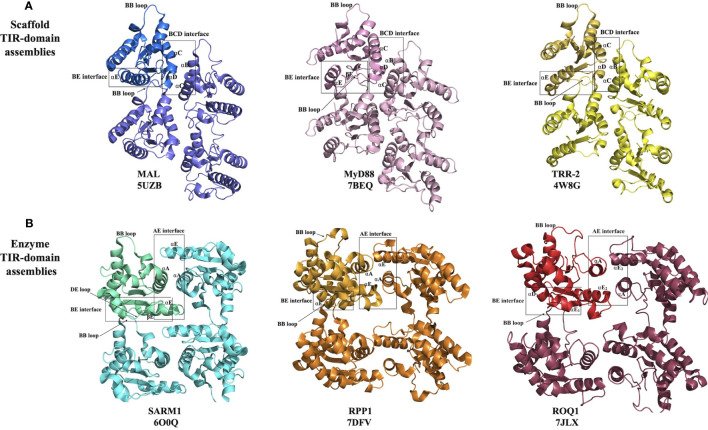
Structural basis of eukaryotic TIR-domain assembly formation. **(A)** Scaffold TIR-domain assemblies, held together by the BE and BCD interfaces, represented by the TIR domains from MAL, MyD88 and TRR-2. **(B)** Enzyme TIR-domain assemblies held together by the BE and AE interfaces, represented by the TIR domains from SARM1, RPP1 and ROQ1.

**Figure 4:**
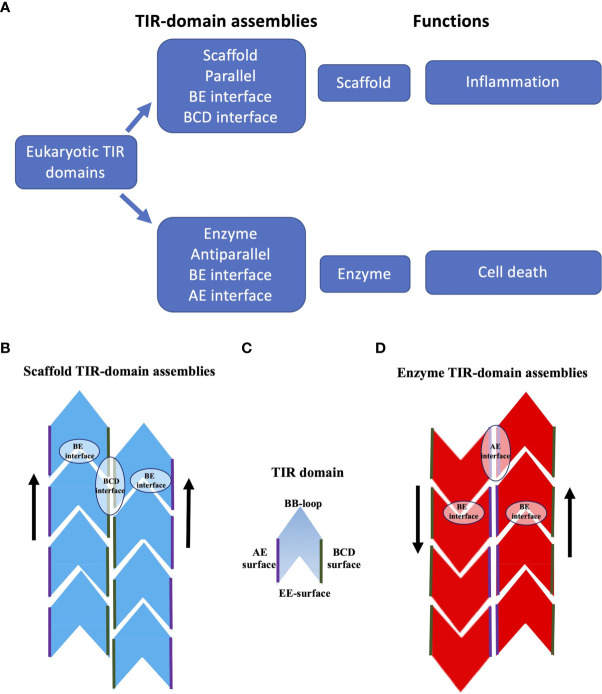
Schematic representation of TIR-domain assemblies. **(A)** Overview of TIR-domain assemblies. **(B)** Scaffold TIR-domain assemblies, represented by the structures formed by the TIR domains from human MAL and MyD88. These parallel two-stranded assemblies are held together by the intrastrand BE interfaces, and the interstrand BCD interfaces. **(C)** Schematic diagram of a single TIR domain, to highlight the BB-loop, BCD, EE and AE surfaces (the diagram does not differentiate the structures of TIR domains in monomeric and signalosome forms). **(D)** Enzyme TIR-domain assemblies, represented by the structures formed by the TIR domains from human SARM1 and the plant NLRs ROQ1 and RPP1. These antiparallel two-stranded assemblies are held together by the intrastrand BE interfaces and the interstrand AE interfaces.

Reconstitution of higher-order assemblies of MAL and MyD88 TIR domains yielded filamentous and micro-crystalline complexes, respectively (26, 27). These reconstitution experiments correlated with the functional signaling pathway, as the TLR4 TIR domain seeded the assembly of MAL TIR domains, while MAL TIR domains seeded the assembly of MyD88 TIR domains. Structure determination of these higher-order assemblies, combined with mutagenesis and signaling assays, has provided clarity on the biologically relevant association of scaffold TIR domains (26, 27). These structures feature two parallel strands of TIR domains, each showcasing a head-to-tail arrangement of TIR domains, held together through a BB-loop-mediated “BE” intrastrand interface. The two strands are offset and held together through a “BCD” interstrand interface (**Figures 3**, **4**).

The structure of the filament formed by MAL TIR domains was determined by helical reconstruction cryo-electron microscopy (cryo-EM) (26). It revealed a hollow tube consisting of 12 protofilaments, with each protofilament corresponding to a two-stranded assembly of TIR domains described above. The intrastrand BE interface includes the area around the BB-loop of one subunit and the EE surface (the βD and βE strands and the αE helix) of the interacting subunit (**Figure 3A**). The interstrand interaction connecting the two strands involves the residues on the BC surface (αB and αC helices) of one subunit from strand 1 and the CD surface (αD helix and the CD loop) of another subunit from strand 2 (**Figure 3A**). Structure-based mutagenesis studies confirmed that key residues in both interfaces (e.g., P125A in the intrastrand interface; L162A, L165A, W156A, Y159A and F193A in the interstrand interface) are necessary for the activation of downstream signaling through NF-κB, whereas mutations of residues mediating interactions between protofilaments were not found to have consequences for function (26).

The structure of the MAL TIR domain-nucleated microcrystals of MyD88 TIR domain was determined using two approaches, microcrystal electron diffraction (microED) and serial femtosecond crystallography (SFX) with an X-ray free electron laser (X-FEL) source (both yielding nearly identical structures) (27). The structure shows that the TIR domains of MyD88 assemble in a fashion analogous to MAL TIR-domain protofilaments (**Figure 3A**). The interfaces responsible for this association were again found to be relevant to signaling, with the mutations R196A, W284A, I253D and R288A in the intrastrand interface, and mutations K238A, L241A, F270A and F270E in the interstrand interface abolishing TLR4-induced NF-κB activation (27). The analogous structural arrangement of TIR domains in the MAL and MyD88 higher-order assemblies suggests a hierarchical, nucleation-controlled and cooperative mechanism for TLR signal transduction, in which the receptor and adaptor TIR domains assemble *via* the inter- and intrastrand interactions observed in the MyD88 and MAL TIR-domain higher-order assemblies, leading to formation of a TIR-domain signalosome. This in turn promotes clustering of the MyD88 death domains (DDs) to form a signalosome termed the “Myddosome” (47), recruiting and activating IRAKs (26, 27), thereby facilitating signaling through a SCAF mechanism.

Several mutations in the TIR domains of TLRs and adaptors are associated with disease. In MyD88, the R196C polymorphism, which is associated with susceptibility to pyogenic bacterial infection during childhood (48), maps to the intrastrand interface of the TIR-domain signalosome. Similarly, the L252P gain-of-function variant of MyD88, which is found in diffuse large B cell lymphoma and promotes tumor survival through enhanced NF-κB activation (49), also maps to the intrastrand interface. The corresponding mutant forms extremely stable oligomers, compared to the wild-type protein, explaining the molecular basis of its phenotype (50).

Interestingly, in one of the crystal structures of the TRR-2 TIR domain from the lower metazoan *Hydra magnipapillata* (PDB ID 4W8G), a parallel two-strand arrangement analogous to MAL and MyD88 TIR-domain signalosomes is observed (**Figures 1**, **3A**). These signalosomes are therefore likely to be structurally conserved in a range of eukaryotes.

The interactions observed in the MyD88 and MAL TIR-domain signalosomes were not captured in the crystal structures of any of the mammalian TLR or adaptor TIR domains. However, surfaces equivalent to the ones mediating interstrand interactions (involving residues in αB, αC and αD helices and the BB-loop; “BCD surface”) are found to mediate symmetric dimer formation in the crystals of a number of TLR and IL-1R TIR domains (TLR1, TLR2, TLR6, TLR10 and IL-1R9) (5). While it is possible that functional surfaces simply find alternative, biologically irrelevant binding partners when forming crystals, it could be that such symmetric interaction contributes to the regulation of signaling by representing an inactive, auto-inhibited state, especially in the case of TLRs that always exist as dimers; such an interaction would presumably be broken when the receptor is activated by ligand binding and the signalosome formation would be allowed.

In summary, the scaffold TIR-domain signalosomes correspond to parallel two-stranded open-ended assemblies of TIR domains, held together by two asymmetric interactions, the intrastrand and interstrand interactions, mediated by the BE and BCD interfaces, respectively (**Figures 3**, **4**). The nucleation-controlled cooperative assembly of these signalosomes is responsible for the SCAF mechanism of signaling.

## Enzyme TIR-Domain Assemblies

This group comprises the TIR domains that undergo self-association, which facilitates NAD^+^-nucleosidase activity and eventually cell death. It includes TIR domains from the mammalian protein SARM1 and plant TIR domain-containing proteins. While some bacterial and archaeal TIR domains have also been shown to have NAD^+^-nucleosidase activity, the structural basis of their self-association and enzymatic activity is not well characterized and may be different from their eukaryotic counterparts discussed in this group (10, 20). On the other hand, the TIR domains from SARM1 and plant NLRs assemble in an analogous fashion, forming two-stranded assemblies different from that of the scaffold TIR-domain assemblies (**Figures 3**, **4**). The association of TIR domains in each individual strand resembles the one observed in scaffold TIR-domain signalosomes, featuring a head-to-tail arrangement mediated by the BB-loop-containing BE interface. However, the two strands in enzyme TIR-domain assemblies are associated in an antiparallel, rather than parallel, fashion. The interstrand interface corresponds to a symmetric “AE interface”, involving the αA and αE helices. This structural information is based on three key structures: the crystal structure of the SARM1 TIR domain (10), and the cryo-EM structures of activated resistosome complexes of the TNLs ROQ1 and RPP1 (15, 16).

### SARM1

While SARM1 has a number of suggested roles in the regulation of innate immunity, the central function appears to be to serve as the executioner of Wallerian or programmed axon degeneration, a highly conserved pathway of injury-induced axon degeneration (51–54). SARM1 facilitates rapid depletion of NAD^+^ in response to axon injury, leading to subsequent axon demise (10, 11, 55). SARM1 deletion mutants lacking the TIR domain show a dominant-negative phenotype, delaying axon degeneration (52). In addition to its C-terminal TIR domain, SARM1 contains an N-terminal ARM (armadillo repeat motif) domain and central tandem SAM (sterile alpha motif) domains. SAM domains form an octameric ring, and the formation of such an oligomeric state is essential for axon degeneration (10, 52, 56). The SARM1 TIR domain has intrinsic NAD^+^-nucleosidase activity, cleaving NAD^+^ into nicotinamide and either ADPR (ADP-ribose) or cyclic ADPR (cADPR) (10, 11, 57). The active site has similarities to that of the NAD^+^ glycohydrolase CD38, including a catalytic glutamate residue (10, 11). Mutation of this residue is sufficient to abolish NAD^+^-nucleosidase activity, indicating similar mechanisms of NAD^+^-nucleosidase activity among these enzymes (10, 11). Self-association of SARM1 TIR domains is essential for enzyme activity (10, 11). The crystal structure of the SARM1 TIR domain revealed the two-stranded antiparallel arrangement described above, and mutational analysis of the BE (D594A, E596K, and G601P) and AE interfaces (L579A and H685A) revealed that these interactions are functionally important for stabilization of the active conformation of the TIR domains (10).

In the inactive SARM1 octamer, interaction of the ARM and TIR domains is responsible for preventing TIR-domain self-association, directly inhibiting NAD^+^-nucleosidase activity (58–60). The ARM-TIR inhibitory interaction is regulated by the cellular ratios of NAD^+^ and its metabolites NMN (nicotinamide mononucleotide) and NaMN (nicotinic acid mononucleotide), through binding to an allosteric site in the ARM domain; NMN as an activator, and NAD^+^ and NaMN as inhibitors (59–63). Injury-associated increase in NMN results in the release of this ARM-TIR autoinhibition and subsequent assembly of TIR domains (59).

### Plant TIR Domains

TIR domains from several plant NLRs and TIR-only plant proteins can also cleave NAD^+^, dependent on the TIR domain self-association and the conserved glutamate residue at the catalytic site; this activity is essential for cell-death signaling (10, 14). Crystal structures of numerous plant TIR domains [including those from RPS4 and RPS4-RRS1 heterodimer, AtTIR, SNC1, RPP1 and RPV1 (64–67)] feature a symmetrical AE interface analogous to the one observed in SARM1, and mutagenesis of residues in this interface was shown to abolish cell-death signaling in plants (10, 65–68). The AE interface often features conserved residues, such as the SH (serine-histidine) motif in the αA helices of the TIR domains, which allows stacking and hydrogen-bonding interactions across the interface (65). Charged residues surrounding the SH motif further stabilize the AE interface (67). However, the structure of a functional TIR-domain assembly only became clear based on the cryo-EM structures of ROQ1 and RPP1 NLRs (15, 16).

Activated ROQ1 and RPP1 form tetrameric structures, largely through the interactions of their NBDs (15, 16). This NBD-mediated tetramer exhibits four-fold symmetry; however, the TIR domains arranged on top of this tetramer exhibit only two-fold symmetry, featuring a dimer of dimers. The arrangement of TIR domains is identical to the arrangement of SARM1 TIR domains observed in the corresponding crystals. Therefore, each dimer features the symmetrical AE interface, but the two dimers are held together through BE interfaces (**Figure 3B**). Such a BE interface was never observed in any of the crystal structures of plant TIR domains; however, the DE surface mediating this interaction has been implicated previously in the function of these domains through site-directed mutagenesis (67–69).

The BE interface-mediated interaction is crucial for NAD^+^-nucleosidase activity, because in the absence of this interaction, the positively charged lysine and arginine residues in the BB-loop block NAD^+^ binding to the active site. In the presence of BE-interface interaction, the BB-loop moves under the DE surface and allows access by the substrate to the NAD^+^-binding site. Jointly, the AE and BE interface-mediated interactions facilitate the appropriate conformation of the active site to enable enzymatic activity (16). The oligomerization of plant NLRs, driven largely by the NBDs, is presumed to nucleate assembly of the TIR domains.

In summary, the enzyme TIR-domain signalosomes involve anti parallel head-to-tail asymmetric intrastrand and symmetric interstrand interactions, mediated by the BE and AE interfaces, respectively (**Figures 3**, **4**). Such an assembly is required for configuring the NAD^+^-nucleosidase active site, and consequently enzymatic activity and biological function (axon degeneration or cell death).

## Signaling by Cooperative Assembly Formation

Both the scaffold and enzyme types of TIR-domain signalosomes function through a signaling mechanism termed SCAF (5–7). Compared to a more gradual signal amplification in a classical signaling pathway, SCAF enables a rapid and strong response to minute amounts of stimulus, resembling a switch; it is therefore well suited to innate-immunity and cell-death pathways. An important aspect of regulation involves the nucleation barriers to oligomerization (25). In the case of scaffold TIR-domain assemblies, the effector enzymes correspond to proteins kinases (IRAKs), which can activate themselves through phosphorylation when brought together. In the case of enzyme TIR-domain assemblies, the TIR domains themselves serve as effector enzymes, as they require self-association for activity. In both scaffold and enzyme groups, other domains play key roles to create a functional pathway, for example LRR domains as receptors (also called sensors) in TLRs and plant NLRs, ARM domains as receptors in SARM1, and DDs and NBDs as adaptors in TLR and plant NLR pathways. The presence of more than one self-associating domain provides further opportunities for regulation of the system (e.g. auto-inhibition between the DD and TIR domains in MyD88), which ultimately creates a large range of concentrations where the system is poised for activation (50, 70, 71).

## Conclusions and Future Directions

TIR domains are found in animals, plants, bacteria and archaea, and often have functions associated with innate immunity and cell death. Self-association of TIR domains is key to their function, but weak affinities prevent association until aided by activating ligands, other domains in TIR domain-containing proteins, or adaptor proteins. The differences in the structures of scaffold and enzyme TIR-domain assemblies correlate with their distinct functional roles. Scaffold TIR-domain signalosomes, represented by MAL and MyD88 TIR-domain complexes, correspond to parallel two-stranded assemblies that are used in nucleation-controlled pathways that are activated by PAMPs or DAMPs and result in the activation of protein kinases (such as IRAKs). Enzyme TIR-domain signalosomes, represented by TIR-domain complexes from SARM1 and plant NLRs, correspond to antiparallel two-stranded assemblies with NAD^+^-nucleosidase activity that form in response to NMN or plant pathogen effector protein binding, respectively. Both groups of assemblies feature an analogous head-to-tail arrangement of TIR domains in individual strands, mediated by the BE interface, but differ in their interstrand association. In line with their analogous interstrand interactions, the functionally important BB-loop adopts a similar conformation in the assembled state in both cases.

Our classification into the two groups of TIR-domain signalosomes is based on a limited number of signalosome structures, and further work will be required to establish how generally applicable the current models are to TIR-domain signaling, and what variations exist in different pathways. However, in support of our general conclusions, molecular, mutational, and functional data on a large variety of TIR domains are consistent with our proposed models. For example, the TIR domain of the TLR adaptor TRAM only has limited sequence identity to those from MAL and MyD88, yet it is compatible with filaments with the same arrangement of TIR domains as seen in the latter two cases (26). Similarly, the *Arabidopsis* plant TIR-only immune receptor RBA1 and the TIR-only protein BdTIR of unknown function from the monocot plant *Brachypodium distachyon* display self-association-dependent NAD^+^-nucleosidase activity (14). Nevertheless, further studies on different TIR domain-containing proteins will be required to understand the breadth of similarities and differences in their molecular basis of function, in particular the less characterized proteins from bacteria and archaea. Another important but poorly characterized aspect involves the molecular details of the reaction catalyzed by different enzyme TIR domains. While all characterized enzyme TIR domains cleave the nicotinamide moiety off the substrate NAD^+^ molecule, many produce a cyclic variant of the remaining ADPR, rather than just the linear ADPR molecule. Human SARM1 produces a small proportion of the classical cADPR, but several bacterial and plant TIR domains instead produce variants with a different cyclic linkage (11, 14, 20). The chemical structures of these variants remain uncharacterized, as do their roles in the corresponding biological pathways.

## Author Contributions

All authors contributed to the article and approved the submitted version.

## Funding

This work was supported by funding from the National Health and Medical Research Council (NHMRC) (Project Grant 1160570 to BK) and the Australian Research Council (ARC) (Discovery Project DP190102526 and Laureate Fellowship FL180100109 to BK).

## Conflict of Interest

BK is a shareholder of Disarm Therapeutics, a wholly owned subsidiary of Eli Lilly & Company. BK is a consultant to Disarm Therapeutics. BK and WG receive research funding from Disarm Therapeutics.

The remaining authors declare that the research was conducted in the absence of any commercial or financial relationships that could be construed as a potential conflict of interest.

## Publisher’s Note

All claims expressed in this article are solely those of the authors and do not necessarily represent those of their affiliated organizations, or those of the publisher, the editors and the reviewers. Any product that may be evaluated in this article, or claim that may be made by its manufacturer, is not guaranteed or endorsed by the publisher.
